# Mitotic Arrest in Teratoma Susceptible Fetal Male Germ Cells

**DOI:** 10.1371/journal.pone.0020736

**Published:** 2011-06-02

**Authors:** Patrick S. Western, Rachael A. Ralli, Stephanie I. Wakeling, Camden Lo, Jocelyn A. van den Bergen, Denise C. Miles, Andrew H. Sinclair

**Affiliations:** 1 Centre for Reproduction and Development, Monash Institute of Medical Research, Monash University, Clayton, Victoria, Australia; 2 Cancer Therapeutics Program, Peter MacCallum Cancer Centre, East Melbourne, Victoria, Australia; 3 Monash Micro Imaging, Monash Health Research Precinct, Monash Institute of Medical Research, Monash University, Clayton, Victoria, Australia; 4 Early Development and Disease, Murdoch Childrens Research Institute, University of Melbourne Department of Paediatrics, Royal Children's Hospital, Parkville, Victoria, Australia; University of Southern California, United States of America

## Abstract

Formation of germ cell derived teratomas occurs in mice of the 129/SvJ strain, but not in C57Bl/6 inbred or CD1 outbred mice. Despite this, there have been few comparative studies aimed at determining the similarities and differences between teratoma susceptible and non-susceptible mouse strains. This study examines the entry of fetal germ cells into the male pathway and mitotic arrest in 129T2/SvJ mice. We find that although the entry of fetal germ cells into mitotic arrest is similar between 129T2/SvJ, C57Bl/6 and CD1 mice, there were significant differences in the size and germ cell content of the testis cords in these strains. In 129T2/SvJ mice germ cell mitotic arrest involves upregulation of p27^KIP1^, p15^INK4B^, activation of RB, the expression of male germ cell differentiation markers NANOS2, DNMT3L and MILI and repression of the pluripotency network. The germ-line markers DPPA2 and DPPA4 show reciprocal repression and upregulation, respectively, while FGFR3 is substantially enriched in the nucleus of differentiating male germ cells. Further understanding of fetal male germ cell differentiation promises to provide insight into disorders of the testis and germ cell lineage, such as testis tumour formation and infertility.

## Introduction

Development of the spermatogenic and oogenic lineages is initiated from bi-potential primordial germ cells in the mid-gestation mouse fetus. Commitment to male or female germ cell development is dependant not on the chromosomal sex of the germ cells, but on the surrounding gonadal cell environment indicating that intercellular signalling plays a central role in this process (For reviews see [1], [2], [3], [4]). Commitment of the germ cells to male development occurs by E12.5 and results in a period of mitotic quiescence. The male germ cells then re-enter the cell cycle post-natally and undergo a highly regulated phase of spermatogenic differentiation. Female germ cell commitment occurs at E13.5 and the developing oocytes immediately enter the meiotic program, arrest in diplotene at birth and ultimately complete meiosis during oocyte maturation [5].

The signals regulating fetal germ cell commitment are poorly understood. Studies over the past five years indicate that retinoic acid (RA) acts in concert with *Dazl* (Deleted in azoospermia-like) to activate *Stra8* (Stimulated by retinoic acid gene 8) and promote entry of female germ cells into meiosis [6], [7], [8], [9]. Furthermore, FGF9 is required for male germ cell survival and it has been postulated that the balance between *Fgf9* (Fibroblast growth factor 9) transcription and RA influences male/female germ cell fate [10], [11]. However, recent studies question the role of RA in these processes [12], [13].

Once committed to male development the germ cells enter a period of mitotic arrest that is tightly associated with expression of the G1-S phase regulators p27^KIP1^ (*Cdkn1b*; cyclin-dependent kinase inhibitor 1B), p15^INK4B^ (*Cdkn2b*; cyclin-dependent kinase inhibitor 2B) and p16^INK4A^ (*Cdkn2A*; cyclin-dependent kinase inhibitor 2A) and the activation of the key G1-S phase check point protein RB (Retinoblastoma) [14]. Consistent with this, loss of RB function leads to disrupted germ cell mitotic arrest, however, this phenotype resolves by embryonic day (E)16.5 at which time levels of *p27^Kip1^* and *p15^Ink4b^* transcription are abnormally high in *Rb* null gonads [15]. Although essentially all male germ cells enter mitotic arrest between E12.5 and E14.5 in mice of a mixed C57Bl/6-CD1 background, some variation in the timing of mitotic arrest is evident between the pure C57Bl/6 and CD1 strains [14].

Previously, the period of male germ cell commitment and entry into mitotic arrest has been associated with susceptibility to teratoma formation. Leroy Stevens showed that spontaneous germ cell derived testicular teratomas occur only in mice of the 129/SvJ strain [16], [17]. In addition, incidence of teratoma formation was substantially reduced between E12.5 and E14.5 and this reduction was dependent on the age of the germ cells and to some extent, the surrounding somatic cells [16], [18]. Furthermore, sub-strains forming testicular teratomas at a high rate exhibit a longer period of proliferation than those forming teratomas at a lower frequency [19]. The genetic and environmental conditions that confer teratoma susceptibility in the 129/SvJ background remain poorly understood, although mutations in several genes including *Dnd1* (Dead-end 1), *Dmrt1* (Doublesex and mab-3 related transcription factor 1), *Pten* (Phosphatase and tensin homolog) and *Bax* (Bcl2-associated X protein) underlie increased tumour incidence [20], [21], [22], [23], [24].

As germ cells migrate and populate the developing mouse gonads they strongly express the core regulators of pluripotency, *Oct4* (Octamer binding protein 4, officially known as Pou5F1, POU domain, class 5, transcription factor 1), *Sox2* (SRY-box containing gene 2) and *Nanog* (Nanog homeobox) and it is possible to use germ cells to establish pluripotent cell cultures until E12.5. After E12.5 the ability of germ cells to form pluripotent cell lines is diminished, the germ cells enter mitotic arrest, the pluripotency factors OCT4, *Sox2*, *Nanog* are repressed and male differentiation genes, such as *Nanos2* (Nanos homolog 2) and *Dnmt3l* (DNA cytosine-5-methyltransferase 3-like), are activated [25], [26], [27], [28], [29], [30], [31]. The timing of these events correlates with the loss of the ability of fetal male germ cells to form spontaneous germ cell tumours in 129/SvJ mice. Furthermore, germ cells forming teratomas in mice fail to appropriately regulate cell cycle and pluripotency and in some cases have a greater propensity to form EG cells *in-vitro* [20], [21], [22], [23].

In order to compare early male germ line development between mouse strains that are susceptible and non-susceptible to spontaneous testicular teratoma formation we compared the entry of germ cells in the 129T2/SvJ strain, specifically isolated by Stevens for its ability to spontaneously form testicular teratomas, with that of the testis tumour non-susceptible strains CD1 (outbred) and C57Bl/6 (inbred) using flow cytometry. We find that although differences in the timing of mitotic arrest are apparent between the inbred and outbred strains, germ cells in the 129T2/SvJ and C57Bl/6 strains enter mitotic arrest at the same developmental time. In addition, the activation of cell cycle regulators p27^KIP1^, p15^INK4B^, p16^INK4A^ and RB and repression of KI67 is co-incident with mitotic arrest in 129T2/SvJ mice. Furthermore, the pluripotent markers OCT4, SOX2 and DPPA2 are down-regulated either during, or immediately after 129T2/SvJ male germ cells enter mitotic arrest. Interestingly, as DPPA2 is down-regulated DPPA4 protein expression substantially increases. Consistent with this, expression of the markers of male germ cell differentiation MILI (also known as PIWIL2; piwi-like homolog 2), NANOS2 and DNMT3L are activated during or immediately after germ cells enter mitotic arrest. Finally, we detected a striking difference between the 129T2/SvJ, C57Bl/6 and CD1 strains in the relative cross sectional size and germ cell content of the testis cords. These differences may affect the susceptibility of germ cells in the 129T2/SvJ, CD1 and C57Bl/6 strains to form teratomas.

## Results

**Figure 1:**
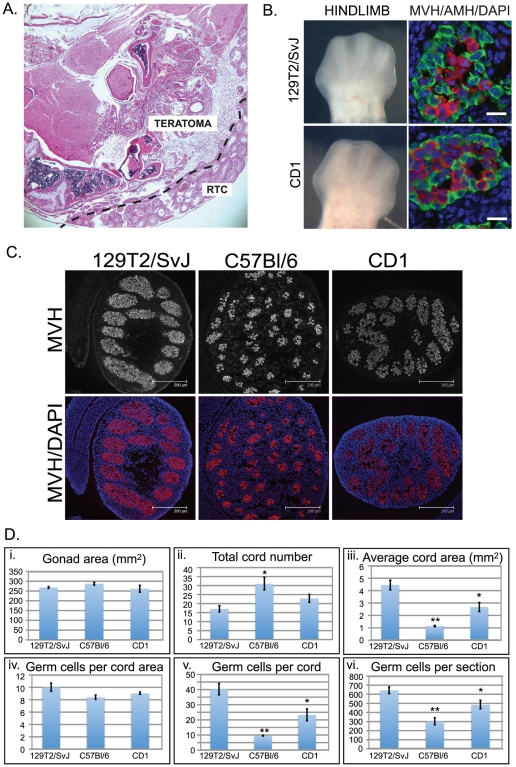
Developmental characteristics of 129T2/SvJ mouse gonads. A. H&E stained cross section of a teratoma collected from an adult 129T2/SvJ mouse testis. RTC: residual testis cords. B. Bright field images showing hindlimb morphology of typical E12.5 129T2/SvJ and CD1 embryos (Left images). Immunofluorescent staining of E12.5 fetal testis sections from 129T2/SvJ and CD1 mice. AMH is shown in green, while MVH is shown in red. DAPI (blue) marks the cell nuclei. Scale bars; 10 um. C. Immunofluorescence showing the typical extent of the germ cell populations marked by MVH staining (red) enclosed by testis cords in E15.5 129T2/SvJ, C57Bl/6 and CD1 fetal testes (a further four independent individuals are shown in Figure S1). DAPI (blue) marks the cell nuclei. Scale bars; 200 um. D. Quantification of the gonad area, total cord number, average cord area, number of germ cells per cord area, the number of germ cells per cord and the number of germ cells per section for whole testis sections taken from E15.5 129T2/SvJ, C57Bl/6 and CD1 embryos (Data represents +/−SEM, eight-ten sections for at least 4 testes). Statistical differences compared to 129T2/SvJ were calculated: * indicates p<0.05, ** indicates p<0.01.

Initially we monitored the 129T2/SvJ males in our colony to determine whether they retained a propensity to spontaneously form teratomas. Examination of the size and shape of testes dissected from 68 129T2/SvJ adult males indicated that teratomas were present in 6 individuals and that there was an unusually high number of significantly under-developed (approximately 2–3 mm in length) left-hand testes (5 individuals). Histological examination of the testes from the 6 individuals that potentially contained teratomas revealed the presence of well-developed teratomas in each case (Figure 1a), confirming that the male mice in our 129T2/SvJ colony are susceptible to relatively common spontaneous teratoma formation. By contrast, and consistent with previous observations, teratomas have not been observed in either CD1 or C57Bl/6 males in our facility.

To compare the progression of mitotic arrest in different strains we collected E12.5-E15.5 embryos from pure CD1, C57Bl/6 and 129T2/SvJ matings. During the collection of samples we observed that the developmental rate and size of embryos was different between the strains. CD1 embryos collected around mid-day, typically contained 26-28 tail somites and exhibited foot-plate morphology consistent with E12.5 staging [32]. By contrast, both 129T2/SvJ and C57Bl/6 embryos collected at a similar time were smaller than CD1 embryos with less tail somites (approximately 24–26) and more rounded forelimb and hindlimb footplate morphology typical of earlier E12.5 embryos. We therefore collected the 129T2/SvJ and C57Bl/6 embryos later in the afternoon (typically around 4 pm). This strategy allowed collection of embryos of a comparable stage (26–28 tail somites) to the E12.5 CD1 embryos (Figure 1b).

In order to confirm that testis development had initiated and the testis cords had enclosed the germ cells by E12.5 we stained testis sections from CD1, 129T2/SvJ and C57Bl/6 embryos with antibodies specific for the Sertoli cell markers SOX9 (SRY box containing gene 9) and AMH (anti-müllerian hormone) and the germ cell marker MVH (mouse vasa homologue, officially known as DDX4; DEAD (Asp-Glu-Ala-Asp) box polypeptide 4). As expected SOX9 and AMH positive Sertoli cell had organised around MVH positive germ cells, confirming the formation of well-developed cords in each strain (Figure 1b, AMH only shown).

We also examined the cord structure in the testes of the different mouse strains at E15.5, by which time fetal testis development is well advanced. In 129T2/SvJ testes there were significantly fewer cords that were comparatively larger and contained more germ cells than in the C57Bl/6 testes. By contrast the cords of C57BL/6 testes were smaller, more numerous and contained relatively few germ cells. Interestingly, the cord size and germ cell content in the CD1 strain fell between that observed for 129T2/SvJ and C57Bl/6 (Figure 1c, 1d, Figure S1). These differences were apparent despite there being no significant difference in testis cross sectional area or the number of germ cells per cord area (Figure 1d i, iv). Thus, although at E12.5 the testes of the different strains all contained well-organised cords, by E15.5 significant strain differences were noted in the number and size of the testis cords and the number of germ cells they contained.

**Figure 2:**
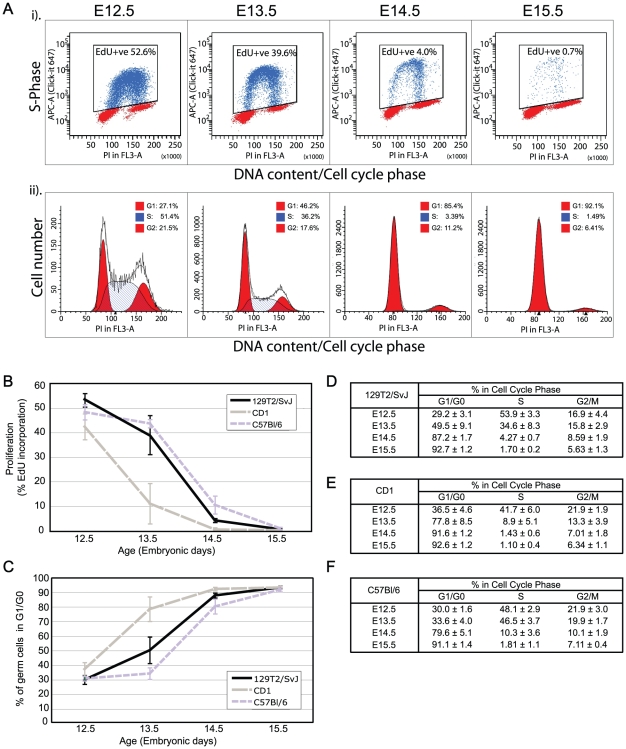
Fluorescence activated flow cytometric analysis of S-phase progression and cell cycle state in teratoma susceptible and non-susceptible fetal mouse germ cells. (A) (i) Typical example of E12.5-E15.5 129T2/SvJ germ cells analysed for EdU incorporation after 2 hours of *in-vivo* exposure to EdU. MVH positive cells were isolated against an MVH stained limb control (MVH negative) sample ([14], not shown) while the EdU gate was set against an EdU negative control. (ii) ModFit cell cycle analysis based on DNA content assessed by propidium iodide staining in the same germ cell populations as shown in (i). (B) Germ cell proliferation based on EdU incorporation in E12.5-E15.5 129T2/SvJ, C57Bl/6 and CD1 fetal gonads. Data is represented by 3-6 biological replicates for each time point and strain analysed. Error bars represent standard deviation. (C–F) ModFit analysis of germ cell cycle state, based on propidium iodide staining for DNA content in E12.5-E15.5 129T2/SvJ, C57Bl/6 and CD1 fetal gonads. C: percentage cells in G0/G1 for the 129T2/SvJ, C57Bl/6 and CD1 strains, D–F: percentage of cells in each cell cycle stage for 129T2/SvJ, CD1 and C57Bl/6. Data is represented by 3–6 biological replicates +/− standard deviation for each time point and strain analysed.

We next examined the progression of the male germ cell population into mitotic arrest for each mouse strain. The proportion of cells progressing through S-phase in each sample was determined by exposing the embryos to EdU *in vivo* for two hours, collecting cleanly dissected gonad samples from E12.5, E13.5, E14.5 and E15.5 CD1, 129T2/SvJ and C57Bl/6 embryos and subjecting them to flow cytometric analysis. In addition to EdU, the samples were stained with propidium iodide to determine the DNA content of each cell and facilitate cell cycle analysis using ModFit LT. A typical example of this analysis in the 129T2/SvJ strain is shown in Figure 2a. Although the great majority of male germ cells (∼93%) in each strain entered mitotic arrest between E12.5 and E14.5, some differences were observed in the timing of this process. For example the majority of germ cells in the CD1 mice had accumulated in G1/G0 by E13.5, while in the 129T2/SvJ and C57Bl/6 strains the majority of germ cells entered mitotic arrest by E14.5 (Figure 2b–f). Despite this difference, the rate of mitotic arrest appeared similar in each strain as judged by the rate of decrease in the percentage of germ cells incorporating EdU (i.e. those in S-phase) or the percentage accumulating in G1/G0 over time (assessed by DNA content, Figure 2b–f).

**Figure 3:**
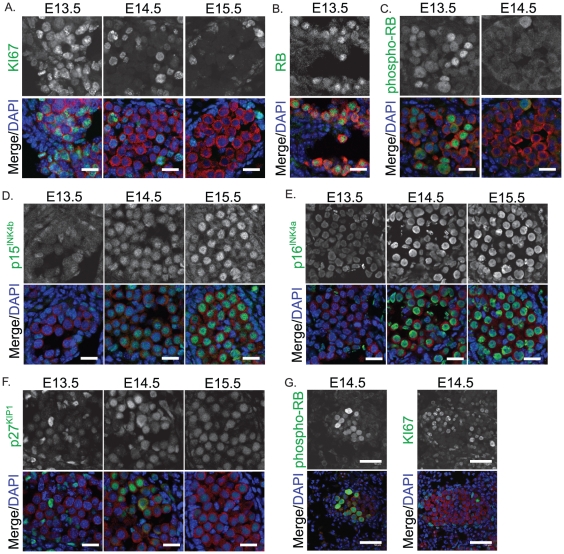
Immunofluorescent staining of cell cycle regulators, KI-67 and retinoblastoma (RB) in E13.5–15.5 fetal testis sections from 129T2/SvJ mice. (A) KI-67 (green) (B) Total RB (green, E13.5 only shown) (C) phosphorylated RB (green, E13.5 and E14.5 shown), (D) p15^INK4b^, (E) p16^INK4a^ and (F) p27^KIP1^ (G) an example of the few cords showing retained expression of phosphorylated RB and KI67 at E14.5. MVH (red) marks the germ cells and DAPI (blue) marks the cell nuclei. Scale bars; 10 um for A–F, 50 um for G.

Previously we have shown that mitotic arrest of germ cells in mice of a mixed CD1-C57BL/6 background involves the repression of KI-67, activation of the cell cycle inhibitors p27^KIP1^, p15^INK4B^, p16^INK4A^ and conversion of phospohorylated (inactive) RB to non-phosporylated (active) RB [14]. Since 129T2/SvJ mice are susceptible to forming spontaneous germ cell tumours we next wanted to determine whether the entry of 129T2/SvJ germ cells into mitotic arrest involved similar cell cycle regulators as were activated in the tumour non-susceptible CD1-C57Bl/6 mice [14]. We therefore stained sections of E13.5, E14.5 and E15.5 129T2/SvJ testes with antibodies specific for KI-67, total RB, phospho-RB, p27^KIP1^, p15^INK4B^ and p16^INK4A^. At E13.5 the germ cell population expressed KI-67 and phospho-RB, but p27^KIP1^, p15^INK4B^ and p16^INK4A^ were not detectable (Figure 3a–g). However, by E14.5 KI-67 and phospho-RB was no longer detected in many germ cells, while p27^KIP1^ and p16^INK4A^ were detected. By E15.5 the majority of germ cells expressed robust levels of p16^INK4A^, p15^INK4B^ and p27^KIP1^ but were negative for KI-67 and phosphorylated RB. Importantly, an antibody that detects both the unphosphorylated and phosphorylated forms of RB revealed RB in germ cells at E13.5 and E14.5, indicating that RB protein was expressed throughout mitotic arrest (Figure 3b, E13.5 only shown). Therefore, 129T2/SvJ germ cells expressed inactive RB prior to arrest while RB was detected in its activated form as germ cells entered mitotic arrest. Interestingly, at E14.5 phospho-RB and KI67 were detected in small groups of germ cells in some cords (Figure 3d). Based on this expression it appeared that these groups of germ cells were entering mitotic arrest later than the majority of the germ cell population. The significance of these groups of late proliferating germ cells remains unknown.

**Figure 4:**
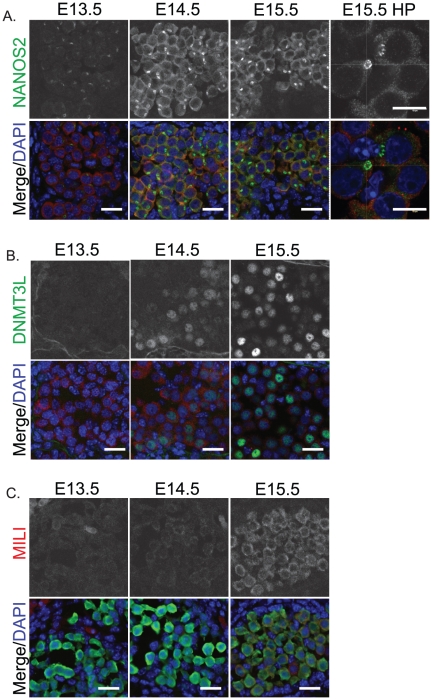
Male fetal germ cell differentiation markers are activated as 129T2/SvJ germ cells enter mitotic arrest. Immunofluorescent staining of (A) NANOS2 (green), (B) DNMT3L (green), and (C) MILI (red) in E13.5–15.5 fetal testis sections from 129T2/SvJ mice. The high power image (HP) (A, far right) shows enrichment of NANOS2 protein to a cytoplasmic body that is largely MVH negative and has a NANOS2 negative core. MVH (red) marks the germ cells in A and B while GFP marks the germ cells in C. DAPI (blue) marks the cell nuclei. Scale bars; 10 um.

We next examined the expression of NANOS2, MILI and DNMT3L, which are involved in fetal male germ cell development [26], [27], [28], [33], [34], [35], [36]. In 129T2/SvJ fetal male germ cells NANOS2 was not detected at E12.5 (not shown), but was detected at low levels within the cytoplasm of E13.5 germ cells and was readily detectable in the cytoplasm of E14.5 and E15.5 germ cells (Figure 4a). Interestingly, NANOS2 protein localised in a strong focal point in E14.5 and E15.5 germ cells. Three-dimensional confocal scanning indicated that NANOS2 delineated the outer edge of a hollow, almost spherical, body that was not enriched for MVH (Figure 4a). Previous work has shown that this localisation is to P-bodies, where NANOS2 is thought to regulate target RNAs [37]. MILI protein was not detected above background in E13.5 and E14.5 129T2/SvJ germ cells, but was readily detected in the germ cell cytoplasm at E15.5 (Figure 4b). DNMT3L was initially detected in a subset of E14.5 fetal male germ cells and was detected at higher levels by E15.5. However, in contrast to MILI and NANOS2, which showed very uniform staining patterns across the germ cell population, DNMT3L expression varied considerably with some germ cells expressing very little, while other germ cells contained relatively high levels (Figure 4c).

**Figure 5:**
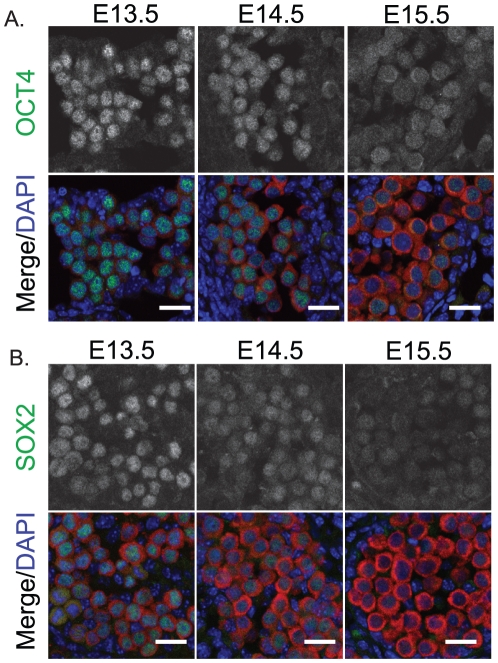
The core pluripotency proteins OCT4 and SOX2 are down-regulated as 129T2/SvJ germ cells enter mitotic arrest. Immunofluorescent staining of (A) OCT4 and (B) SOX2 in E13.5–15.5 fetal testis sections from 129T2/SvJ mice. MVH (red) marks the germ cells and DAPI (blue) marks the cell nuclei. Scale bars; 10 um.

**Figure 6:**
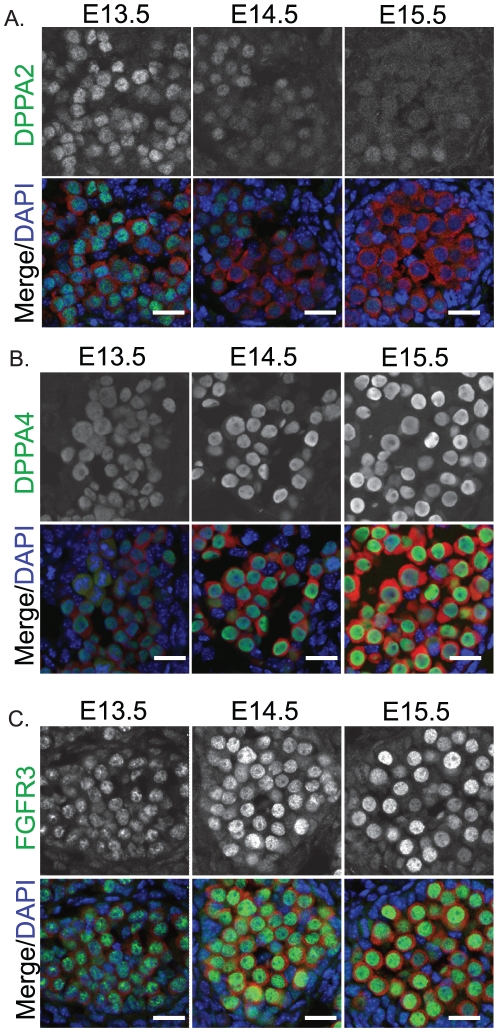
DPPA2 is depleted, while DPPA4 and FGFR3 are enriched in the nuclei of differentiating male germ cells. Immunofluorescent staining of (A) DPPA2, (B) DPPA4 and (C) FGFR3 in E13.5–15.5 fetal testis sections from 129T2/SvJ mice. MVH (red) marks the germ cells and DAPI (blue) marks the cell nuclei. Scale bars; 10 um.

Since pluripotency is normally regulated in male germ cells at these stages, we examined the expression of the core regulators of pluripotency, OCT4 and SOX2, and the pluripotency associated proteins DPPA2 and DPPA4 in fetal germ cells of 129T2/SvJ mice [25], [30]. At E13.5 OCT4, SOX2 and DPPA2 were all expressed in the nucleus at relatively high levels (Figure 5a, b, Figure 6a), while DPPA4 was detected in the nucleus and cytoplasm at low levels (Figure 6b). However, by E14.5 the levels of OCT4, SOX2 and DPPA2 were substantially reduced compared to E13.5 and by E15.5 these proteins were barely detectable (Figure 5a, b, Figure 6a). Although DPPA2 was substantially reduced during E13.5-E15.5, levels of DPPA4 were substantially increased during this period and essentially all DPPA4 protein was now located within the nucleus (Figure 6b).

Finally, since FGF2 is essential for the derivation of pluripotent embryonic germ cells and results in the localisation of FGFR3 to the nucleus of germ cells cultured under these conditions [38], we examined the expression of FGFR3 in fetal germ cells of 129T2SvJ mice as they differentiate down the male lineage. To our surprise FGFR3 was detected at very low levels in E12.5 (not shown) and E13.5 fetal germ cells but was then substantially enriched in the nucleus of germ cells as they entered mitotic arrest between E13.5 and E15.5 (Figure 6c). This nuclear enrichment of FGFR3 was specific to male germ cells, as similar enrichment of FGFR3 was not observed in female germ cells (not shown).

## Discussion

Testicular teratomas form spontaneously in mice of the 129/SvJ strain, but are extremely rare in other inbred strains such as C57Bl/6 or the outbred strain CD1. In 129/SvJ mice the fetal male germ cells are susceptible to forming teratomas at E12.5, however, this susceptibility is rapidly lost during E13.5 and E14.5, implying that entry into the male developmental pathway and mitotic arrest is important in delivering tumour resistance. In this study we have examined the 129T2/SvJ strain, which was originally identified as teratoma susceptible by Leroy Stevens. Although susceptibility to form spontaneous teratomas in this strain is evident (Figure 1a), the mechanisms underlying this susceptibility remain obscure. Comparison of 129T2/SvJ mice with CD1 and C57Bl/6 animals in this study has shown that the germ cell populations enter mitotic arrest with similar dynamics in each strain. Although mitotic arrest occurred relatively earlier in CD1 fetuses compared to those of 129T2/SvJ and C57Bl/6, there was no significant difference in germ cell mitotic arrest between the teratoma susceptible129T2/SvJ strain and the inbred non-susceptible C57Bl/6 strain.

Despite this, there was a striking difference in the size of the E15.5 testis cords and the number of germ cells contained within those cords between 129T2/SvJ and C57Bl/6 mice. In 129T2/SvJ mice, the cords were relatively few, large in size and packed with many germ cells. In contrast, the cords in C57Bl/6 mice were small, more numerous and contained relatively few germ cells (Figure 1 and S1). The increased number of germ cells in the 129T2/SvJ strain could have its origin in any one or a combination of mechanisms: (1) the germ cell cycle may differ slightly between strains such that through several divisions the 129T2/SvJ line accumulates more germ cells; (2) similarly, the rate of cell death could differ slightly between the strains or; (3) the number of germ cells in the founding population at the time of germ cell specification may be different and carried through to this stage of development. Our cell cycle analysis (Figure 2) indicates that the proliferation rate of germ cells in the 129T2/SvJ and C57Bl/6 strains is very similar at E12.5, indicating that proliferation rate may not account for the differences. Moreover, since E12.5-E14.5 C57Bl/6 germ cells proliferate at the same or a sightly higher rate than their 129T2/SvJ counterparts (Figure 2 b, c), it seems unlikely that a greater proliferation rate in 129T2/SvJ than C57Bl/6 during this period can account for the differences in germ cell number. However, it remains possible that subtle or earlier differences in germ cell cycle length or proliferation exist that we have been unable to detect. The possibility that 129T2/SvJ mice might have a greater number of germ cells from early embryonic stages (i.e. at germ cell specification) is of interest. Such a scenario could occur if mice of the 129T2/SvJ strain exhibit an ability to re-establish/retain pluripotent potential in more epiblast cells during germ cell specification, thus founding a larger nascent germ cell population. However, this is speculative and requires investigation.

Interestingly, germ cell tumour formation has been linked both to cell death and to intercellular signalling, which drives germ cell differentiation. Considering this, it is tempting to speculate that the large cords containing numerous germ cells in the 129T2/SvJ mice may confer increased tumour susceptibility. Firstly, death of fetal germ cells through a BAX dependent mechanism has been linked to absence of teratomas in C57Bl/6 mice with mutations in teratoma susceptibility genes. For example mice with mutations in *Dnd1* readily form teratomas in 129/SvJ mice, but do not normally form similar tumours in mice of the C57Bl/6J, LTXBJ or C3H/HeJ backgrounds as the germ cells die. However, *Dnd1* null mice of a mixed 129/SvJ-C57Bl/6 *Bax* null background form teratomas with high frequency indicating that germ cell death is important in preventing teratomas [21]. Secondly, it is well established that the environment of the developing testis is critical for male germ cell differentiation [1], [5], [11], [39], [40]. In human patients with disorders of sex development, testis tumours are frequent and have been linked to the testicular micro-environment [41]. It is possible that in 129T2/SvJ mice, teratoma susceptibility is increased due to the larger cord size and higher germ cell numbers within these cords. This may lead to less efficient inter-cellular somatic-germ cell signalling and decreased activation of male-promoting pathways in some germ cells and/or an increased chance that germ cells escape the cell death program. Interestingly, in E14.5 129T2/SvJ testes we observed some small groups of germ cells that remained positive for the phosphorylated (inactive) form of RB, a mark that strongly indicates they are still proliferative [14], (our unpublished data). Although the significance of these groups remains difficult to assess, the late retention of phosphorylated RB in these cells may be due to their later reception of male signalling molecules.

Despite this, the vast majority of germ cells in 129T2/SvJ mice entered mitotic arrest with very similar molecular dynamics to those of the other two strains examined. The same mitotic arrest proteins were activated in 129T2/SvJ and CD1-C57Bl/6 germ cells. Similarly, activation of NANOS2, MILI and DNMT3L occurred at E14.5–15.5 in 129T2/SvJ mice and the regulators of pluripotency were suppressed with similar timing as other strains [30]. Furthermore, the core pluripotency regulators, OCT4 and SOX2 were also repressed between E13.5 and E15.5 in germ cells of the 129T2/SvJ strain, as we have previously observed in CD1-C57Bl/6 fetal germ cells [30].

We examined two other proteins, DPPA2 and DPPA4 that have previously been associated with pluripotency and germ cell development [25]. Recent evidence supports the conclusion that DPPA4 is required for regulating epigenetic patterns in the early embryo and embryonic stem cells [42], [43]. While the function of DPPA2 remains obscure, expression of this gene is abnormal in various tumours [44]. Analysis of protein levels in this study show that while DPPA2 levels substantially decrease in developing fetal male germ cells of 129T2/SvJ mice, DPPA4 protein levels substantially increase from low levels to high levels during this developmental phase and there is a shift of DPPA4 from the germ cell cytoplasm into the nucleus. The switch from high DPPA2 to high DPPA4 protein levels in the male germ cells of 129T2/SvJ mice coincides with the transition from a period of epigenetic reprogramming in the bi-potential and nascent male gonadal germ cell population (NANOS2, MILI, DNMT3L negative/low) to a period of epigenetic re-establishment in the more differentiated male germ fetal germ line (NANOS2, MILI, DNML3L high). Considering the role of DPPA4 in regulating epigenetic patterns in early embryos and embryonic stem cells [42], [43], the shift in DPPA2/DPPA4 expression in the differentiating male germ line may reflect a role for DPPA4 in regulating the epigenome in the male germ line. The translocation of DPPA4 from the cytoplasm to the nucleus is also of interest as we have previously observed a similar shift in DPPA2 localisation in differentiating cells of inner cell mass outgrowths as they lose pluripotent potential at the outgrowth periphery and lose OCT4 expression [25]. It appears that, in contrast to DPPA2 which is reduced in the nucleus as OCT4 is reduced, DPPA4 becomes enriched in the germ cell nucleus as OCT4 levels drop.

Finally, we have shown that FGFR3 is substantially and sex specifically enriched in the nucleus of fetal male germ cells as they enter mitotic arrest. This is of interest as FGF9 is involved in male germ cell survival and differentiation, and its activity is thought to be mediated through FGFR2 [10], [11]. The data presented here indicates that FGFR3 may play a role in regulating fetal male germ cell differentiation. However, activating mutations in FGFR3 have been linked to the formation of spermatocytic seminomas [45]. Furthermore, FGFR3 becomes enriched in the nucleus of germ cells in the presence of FGF2 and cultured under conditions that allow pluripotent embryonic germ cell formation [38]. Both of these situations apparently lead to less differentiated, rather than more differentiated, “germ” cell types, supporting the conclusion that FGF signalling plays a role in restraining, rather than promoting fetal germ cell differentiation as indicated by the accumulation of FGFR3 in the nucleus of differentiating male fetal germ cells shown here. It is therefore of interest to further clarify the role(s) of FGFR3 in fetal germ cell development.

**Figure 7:**
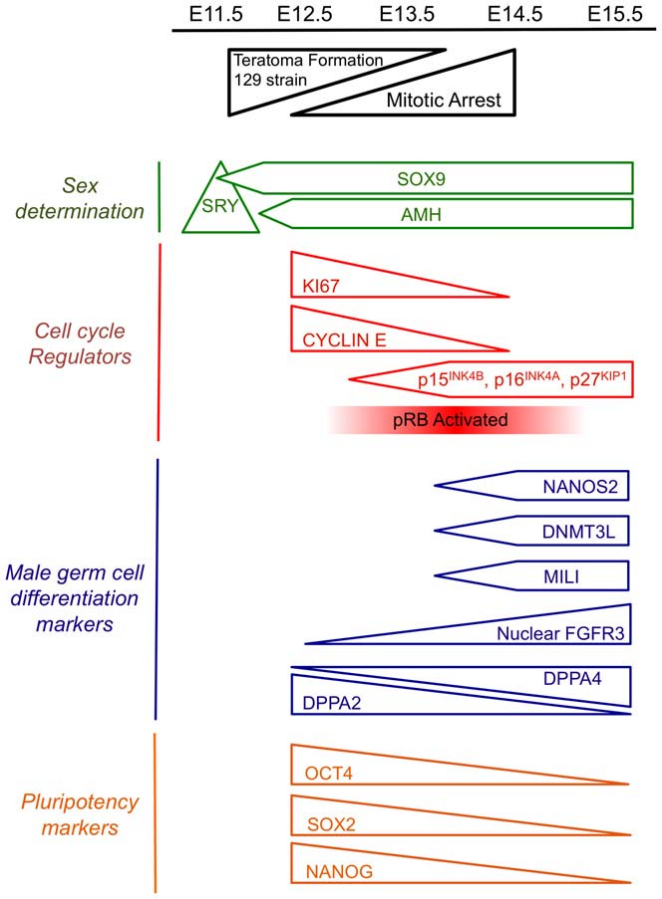
Schematic representation of early male fetal germ cell development. Only mice of the 129T2/SvJ strain form germ cell derived teratomas. Testis determination is initiated by SRY, which activates SOX9 and AMH. Testis cords are formed by E12.5. Germ cells are committed to male development at around E12.5 and enter mitotic arrest between E12.5 and E14.5. This is moderately strain dependent, with germ cells of the outbred CD1 strain entering mitotic arrest slightly earlier than those for the inbred 129T2/SvJ and C57Bl/6 strains. During mitotic arrest the G1 cyclins (particularly *Cyclin E1* and *Cyclin E2*) are repressed, the G1-S cell cycle inhibitors p27^KIP1^, p15^INK4b^, and p16^INK4a^ are upregulated and pRB is activated. Expression of male germ cell differentiation markers including NANOS2, MILI, DNMT3L and FGFR3 increases between E12.5 and E14.5, while the core pluripotency markers OCT4, SOX2 and NANOG are repressed. DPPA2 and DPPA4 exhibit reciprocal expression patterns in E12.5-E15.5 129T2/SvJ male germ cells. This scheme summarises data from the present study, and references 10, 26 and 27.

In this study we have examined the early development of male germ cells in teratoma susceptible 129T2/SvJ mice compared to the non-susceptible strains, C57Bl/6 and CD1. We find that the fetal testes of 129T2/SvJ mice are characterised by few, but large testis cords containing large numbers of germ cells, while fetal testes of the teratoma non-susceptible C57Bl/6 inbred strain are characterised by numerous small cords that contain relatively few germ cells. These differences may underlie increased teratoma susceptibility in the 129T2/SvJ strain. Despite this, mitotic arrest in these strains occurs with similar dynamics and employs similar cell cycle arrest proteins (Figure 7). Since reactivation of pluripotency and misregulation of cell cycle control are characteristics of testis tumours that are derived from fetal germ cell precursors in human patients, further understanding of the molecular regulation of fetal germ cell development promises to impact our understanding of human testis cancers.

## Materials and Methods

### Animals

Mice used in all experiments were derived from CD1-CD1, C57Bl/6-C57Bl/6 or 129T2/SvJ-129T2/SvJ (129, Teratoma 2, Sv Ems Jackson laboratories, Bar Harbor, Maine, USA; referred to here as 129T2/SvJ) matings. Oct4-GFP (OG2) back-crossed for 8 generations mice to 129T2/SvJ mice were used for Figure 4c. All strains were sourced from the Walter and Eliza Hall Institute Animal Facility, Melbourne, Australia. The origin of the 129T2/SvJ mice was the Jackson Laboratories, Bar Harbor, Maine, USA. Mating was detected with the appearance of a vaginal plug in the morning and recorded as E0.5. All animal procedures were carried out under animal ethics permits A619, A622 and A623 issued by the Murdoch Childrens Research Institute Animal Ethics Committee.

### Flow Cytometry

Flow cytometry was performed as previously described with the following variations [14]. Pregnant mice were injected intra-peritoneally with EdU 10 mg/kg and embryos collected after 2 hours. A pool of 8 embryonic gonad/mesonephros samples was dissociated using Trypsin/EDTA and incorporated EdU detected using the 647-Click-iT Flow Cytometry Kit (Invitrogen, Molecular Probes, Carlsbad, CA, USA) with modifications to the protocol as described below. Germ cells were identified by their positive immuno-reactivity with a MVH primary antibody (Abcam, Cambridge, UK) detected using a goat-anti rabbit Alexa Flour 488 secondary antibody (Invitrogen, Molecular Probes, Carlsbad, CA, USA). All washes were performed in 100 ul of the Alexa 647 Click-iT EdU Flow Cytometry kit permwash solution. DNA was stained with 100 ug/ml propidium iodide. Samples containing a minimum of 10,000 germ cells were analysed using an LSRII flow cytometer (BD Biosciences) utilising FACSDiVa software (BD Biosciences). Cell cycle state was assessed on DNA content using ModfitLT (Verity Software House) software. Three to six biological replicates were analysed for each sampling point and mouse strain.

### Immunofluorescence

Gonad samples were collected from E12.5 - E15.5 mouse fetuses and fixed in 4% PFA for 20 min – 1.5 hours depending on age, transferred to 30% sucrose in PBS overnight and frozen in optimal cutting temperature. Sections were cut at 10 µm, permeabilised with 1% Triton-X and non-specific staining blocked with 5% BSA or M.O.M blocking solution (Vector Labs, Burlingame, CA, USA). Primary and secondary antibodies (Table 1) were diluted in 1% BSA or M.O.M dilution buffer. To assess non-specific staining additional sections were analysed using secondary antibody only controls. Where MVH was used with another rabbit raised antibody, the MVH antibody was directly labelled using the Zenon 594 Labelling Kit (Invitrogen Molecular Probes) in accordance with the manufacturer's instructions and staining was performed in 647-Click-iT Flow Cytometry Kit permwash solution. Immunofluorescence was replicated using gonad samples from at least 3 individuals at each sampling point analysed.

**Table 1:** Primary Antibodies.

Protein	Source/Catalogue #	Species	Dilution
AMH	Santa Cruz/sc6886	Goat	1∶200
DMNT3L	Shanghai AntibodyResearch Centre/M014	Mouse	1∶100
DPPA2	Dr Patrick Western	Rabbit	1∶300
DPPA4	R&D Systems/AF3730	Goat	1∶100
FGFR3	Santa Cruz/sc123	Rabbit	1∶200
Ki-67 (SP6)	NeoMarkers/rm-9106-s	Rabbit	1∶50
MVH	Abcam/ab13840-100	Rabbit	1∶10000
NANOS2	Professor Yumiko Saga	Rabbit	1∶100
OCT4	Cell Signalling/2840S	Rabbit	1∶100
p15^INK4B^	Cell Signalling/4822	Rabbit	1∶100
p16^INK4A^	Santa Cruz/sc1661	Mouse	1∶200
p27^KIP1^	Santa Cruz/sc528	Rabbit	1∶100
Rb	BD Biosciences/ 554136	Mouse	1∶50
phosphorylated-Rb (Ser807/811)	Cell Signalling/9308	Rabbit	1∶100
SOX2	Santa Cruz/sc-17320	Goat	1∶50
SOX9	Dr Dagmar Wilhelm	Rabbit	1∶200
MILI	Cell Signalling/2071	Rabbit	1∶200

### Analysis of testis cord size and germ cell content

Fixed gonad sections were stained using our immunofluorescence protocol with MVH or DPPA4 and DAPI and whole testis sections imaged by confocal microscopy. Image J version v1.45d was used to assess the total area of each testis section and each cord, the number of germ cells in each testis section and the number of germ cells in each cord. Cord boundaries were identified by MVH staining of germ cells. The MVH-positive regions were then processed with the following algorithms in order: "Close", "Fill Holes", "Remove Outliers" to achieve an image mask which encompasses only cells with VASA-positive stains. This mask was then applied to the DAPI-stained image with the subsequent algorithms applied in order: "Watershed", "Close", "Fill Holes", "Remove Outliers" to achieve an image containing only the nuclei of VASA-positive cells. The "Analyze Particles" module were then used to count the number of VASA-positive cells. Total tissue area was quantified using a threshold on the upper 88% of DAPI signal intensities and analysed by "Analyze Particles" module. Cord areas were quantified using a threshold for the upper 78% of signal intensities on VASA-stained images and analysed by "Analyze Particle" module. At least eight random sections were analysed from a minimum of four testes for each strain. Standard error was calculated for each data set and statistically significant differences determined. Statistical significance was determined by a two-tailed *t* test assuming equal variance using Microsoft Excel. A p value of less than 0.05 was considered significant. Total cross sectional area for each section and the number of germ cells per cord area was calculated and used as an additional internal control against sampling bias between the strains.

## Supporting Information

Figure S1 Immunofluorescence showing the typical extent of the germ cell populations marked by MVH staining in E15.5 fetal testes from four separate individuals collected from four independent litters each from 129T2/SvJ, C57Bl/6 and CD1 mice. Grey scale image of MVH only shown. Scale bars; 200 um.
(EPS)
